# Dendritic spine morphology and memory formation depend on postsynaptic Caskin proteins

**DOI:** 10.1038/s41598-019-53317-9

**Published:** 2019-11-14

**Authors:** Norbert Bencsik, Szilvia Pusztai, Sándor Borbély, Anna Fekete, Metta Dülk, Viktor Kis, Szabolcs Pesti, Virág Vas, Attila Szűcs, László Buday, Katalin Schlett

**Affiliations:** 1 Department of Physiology and Neurobiology, Eötvös Loránd University, Budapest, Hungary; 2 Institute of Cognitive Neuroscience and Psychology, Research Centre of Natural Sciences, Hungarian Academy of Sciences, Budapest, Hungary; 3 Institute of Enzymology, Research Centre of Natural Sciences, Hungarian Academy of Sciences, Budapest, Hungary; 4 Department Anatomy, Cell and Developmental Biology, Eötvös Loránd University, Budapest, Hungary; 5 Department Medical Chemistry, Semmelweis University, Budapest, Hungary

**Keywords:** Spine plasticity, Long-term potentiation

## Abstract

CASK-interactive proteins, Caskin1 and Caskin2, are multidomain neuronal scaffold proteins. Recent data from Caskin1 knockout animals indicated only a mild role of Caskin1 in anxiety and pain perception. In this work, we show that deletion of both Caskins leads to severe deficits in novelty recognition and spatial memory. Ultrastructural analyses revealed a reduction in synaptic profiles and dendritic spine areas of CA1 hippocampal pyramidal neurons of double knockout mice. Loss of Caskin proteins impaired LTP induction in hippocampal slices, while miniature EPSCs in dissociated hippocampal cultures appeared to be unaffected. In cultured Caskin knockout hippocampal neurons, overexpressed Caskin1 was enriched in dendritic spine heads and increased the amount of mushroom-shaped dendritic spines. Chemically induced LTP (cLTP) mediated enlargement of spine heads was augmented in the knockout mice and was not influenced by Caskin1. Immunocytochemistry and immunoprecipitation confirmed that Shank2, a master scaffold of the postsynaptic density, and Caskin1 co-localized within the same complex. Phosphorylation of AMPA receptors was specifically altered by Caskin deficiency and was not elevated by cLTP treatment further. Taken together, our results prove a previously unnoticed postsynaptic role of Caskin scaffold proteins and indicate that Caskins influence learning abilities via regulating spine morphology and AMPA receptor localisation.

## Introduction

A prime aim of learning is to adapt successfully to an ever-changing environment. Altering the strength of connections between neurons is widely assumed as one of the mechanisms by which memory is encoded and stored in the brain. Activity-dependent long-term synaptic plasticity in adult neural networks also depends on the strengthening or weakening of existing synapses and formation of new contact sites. These effects include structural changes due to altered cytoskeletal dynamics, shape or arborisation^1–4^ or electrophysiological changes in intrinsic membrane properties depending on the type, quantity or localisation of ion channels and neurotransmitter receptors within the plasma membrane^5,6^.

Neuronal scaffold proteins have been reported as molecular hubs important in organizing signalling machinery of both excitatory and inhibitory synapses, regulate actin dynamics or protein transport^7–9^. Within the presynapse, scaffold proteins provide a protein-rich network at the active zone, which is involved in molecular interactions regulating neurotransmitter vesicle docking, priming and exocytosis^10,11^. Neurotransmitter receptor localisation, transport and recycling are also regulated by local scaffold proteins within the postsynaptic site^12^.

CASK-interactive proteins, Caskin1 and Caskin2, are multidomain neuronal scaffold proteins^13^. Caskins are multidomain proteins possessing six ankyrin repeats, a single SH3 domain, two sterile α-motifs (SAM domains) and an extended disordered C-terminal proline-rich domain (PRD)^13^. The N-terminal region shares analogous but evolutionary not conserved domains with Shank proteins, which provide the major scaffolding platform within the postsynaptic density (PSD) of excitatory synapses upon multimerisation. Although Caskin1 and 2 share some structural similarities, they may execute divergent functions due to differences in binding to CASK (Calcium/calmodulin dependent serine protein kinase), the ability for SAM multimerisation and SH3 domain folding^13–15^. Caskin1 is expressed at high levels in the brain, mainly in the synaptic regions^13,16^. Presynaptic localisation of Caskin1 was detected within the ribbon synapses of the bovine retina^17^. Up to date, no data are evident on Caskin2 expression.

Caskin1 is known to bind to several proteins, including Nck/Dock^18^, LAR receptors tyrosine phosphatases^19^ or Abi2^20^. The Caskin name itself is derived from the interaction with CASK^13^. CASK mutations have been widely implicated in neurological diseases, such as microcephaly and X-linked mental retardation^21,22^, but little is known about how the two Caskin proteins, Caskin1 and 2 influence synaptic functions. Nevertheless, Caskin1 has been proposed to play a role in synaptic functions via interacting with CASK protein near the presynaptic plasma membrane^13,17,23^.

In the present study, we investigated how the lack of Caskin proteins affects learning and memory in mice, using double knockout animals for Caskin1 and 2 (Caskin dKO mice). Behavioural analyses and field potential measurements indicated impaired memory and LTP (long-term potentiation) formation in Caskin dKO animals. We also prove that the lack of Caskins and the overexpression of Caskin1 altered dendritic spine morphology of hippocampal neurons in an opposite manner. Analysis of the dendritic protrusions indicates that expression of exogenous Caskin1 significantly increases the ratio of mushroom spines. Importantly, the absence of Caskin proteins augments chemically induced LTP (cLTP) dependent increase in protrusion density and does not influence dendritic spine enlargement in the investigated cultures. Importantly, phosphorylation of the GluA1 subunits of AMPA receptors under normal conditions as well as following cLTP induction was specifically altered in Caskin dKO hippocampal neuronal cultures. These data indicate that Caskins participate in learning and LTP formation via regulating spine morphology as well as influencing AMPA receptor localisation in synapses.

## Materials and Methods

### Study approval

This study was conducted under the approval of the Institutional Animal Ethics Committee of Eötvös Loránd University (approval number: PEI/001/1108-4/2013 and PEI/001/1109-4/2013). All methods were performed in accordance to the guidelines on research ethics of Eötvös Loránd University.

### Animal handling

CD1 (WT), C57Bl6/J (WT), Caskin dKO (Caskin1^−/−^/2^−/−^) and Caskin dHZ (Caskin1^+/−^/2^+/−^) transgenic mice were housed in the animal facility at 22 ± 1 °C, with 12-h light/dark cycles and *ad libitum* approach to food and water. The animals were maintained and handled in accordance with the Guidelines for Accommodation and Care of Animals, according to the European Convention for the Protection of Vertebrate Animals Used for Experimental and Other Scientific Purposes.

### Generation of Caskin dKO mice

Caskin1 constitutive KO mice were generated by targeted disruption of *Caskin1* gene on chromosome 17 by TaconicArtemis. Using the Caskin1 targeting vector, the 2–6 coding exons of *Caskin1* gene were flanked by loxP sites. Neomycin resistance gene cassette was placed into the intron 1 and thymidine kinase gene was inserted next to the homologous sequence for selection markers. Caskin2 constitutive KO mice were produced by targeted disruption of *Caskin2* gene on chromosome 11. Using the Caskin2 targeting vector, the 3–7 coding exons of the *Caskin2* gene were flanked by loxP sites. Neomycin resistance gene cassette was placed into the intron 2 next to the floxed exons.

In both cases, the targeted C57BL/6 N embryonic stem cell lines were grown on a mitotically inactivated mouse fibroblasts feeder layer in DMEM high glucose medium containing 20% FBS and 1200 U/ml LIF. 1 × 10^6^ embryonic stem cells and 30 μg of linearized targeting vector were electroporated (Biorad Gene Pulser) at 240 V and 500 μF. Next, puromycin selection (1 μg/ml; on day 2) and gancyclovir (2 μM; on day 5) counter selection were performed after electroporation. On day 8, embryonic stem cell clones were isolated and analysed by Southern blotting. The identified targeted ES cells were microinjected in blastocysts and transferred to pseudopregnant females. The chimeric mice were bred further and the germline transmission was identified in every generation. The floxed 2–6 exons of *Caskin1* gene or the floxed 3–7 exons of *Caskin2* gene were removed by Cre-mediated recombination, when Cre expressing mouse line (Gt(ROSA)26Sor with C57BL/6J background) was crossed with mice carrying the floxed *Caskin* genes (C57BL/6J background). The knockout-step took place when the Cre enzyme removed the floxed *Caskin1* and *Caskin2* genes in the littermates.

Genotyping was performed by PCR using oligonucleotide primers a1 and s1 (a1: CAAGAGTCCGGTGGACAAGG and s1: ATGTTTCCAGGCCCTCTTGC) for the wild type Caskin1 allele (product size, 306 bp), and oligonucleotides a1 and s2 (s2: CACTGGCTGAACAGCAAAGC) for the exon 2–6 deleted allele (product size, 366 bp). Caskin2 deletion was tested in a second PCR reaction, using primers a2 and s3 (a2: CCTAATGAAGGCACGTCAGG and s3: CACCAACCAACTGCCTTGC) for the amplification of the wild type Caskin2 allele (product size, 255 bp), and primers a2 and s4 (s4: ATAACTCAGTGGTGAAGACAGTGC) for the amplification of the exon 3–7 deleted allele (product size, 315 bp). Inactivation of double (Caskin1 and Caskin2) genes was tested in every generation by PCR of genomic DNA. Caskin dKO mice were obtained by interbreeding Caskin1 KO and Caskin2 KO mice.

### Immunohistochemistry

3 months old C57Bl6/J wild-type (WT) or Caskin dKO mice were deeply anesthetized with chloral-hydrate (350 mg/kg, i.p.) and were transcardially perfused with ice-cold 4% paraformaldehyde (TAAB, wt/vol in PBS; pH 7.4). Following dissection, brains were postfixed overnight in 4% paraformaldehyde and cryoprotected in 30% sucrose in PBS at pH 7.4. Sagittal sections (45-μm thick) of the brain were cut on a cryostat (Leica). Gallocyanine-chrome alum stainings were performed on the sections mounted on gelatine–coated slides. The slides were immersed in the 0.15% gallocyanine-chrome alum solution for 1 day and then washed distilled water for 5 min. After staining, slides were dehydrated in graded ethanol (50%, 70%, 96% and absolute ethanol) and xylene for five minutes in each solution, then and coverslipped with DPX (Merck). Images were taken with a Zeiss AxioObserver Z1 (Carl Zeiss) with Plan Neofluar 10x/0.3 objective.

### Behavioural tests

Animal behavioural experiments were performed essentially as previously described in Bencsik *et al*.^24^. 16 control (Caskin dHZ) and 16 Caskin dKO transgenic littermates were used in behavioural tests. In brief, the open field tests were carried out at three different ages. Mice were placed in the middle of a 48 × 48 × 40-cm open box (Experimetria Ltd.) and were allowed to move freely for 5 min. Their behaviour was recorded and analysed by the Conducta Advanced System 1.0.

Novel object recognition tests were carried out at three different ages^25^. Experiments were carried out within a non-transparent white cage (25 cm × 42 cm). Prior to training, mice were habituated to the cage for 5 min. The next day, animals were introduced into the same field from the previous day but this time the cage contained two identical objects (A1 and A2). Mice were let to explore the environment for 5 min, and the time the animals spent with sniffing objects A1 and A2 was evaluated from the recordings, respectively. 5 h later, mice were re-introduced to the cage, containing one familiar (A1) and a novel (B) object. The mice could explore the cage and the objects again for 5 min. Rodents usually remember the familiar (A1) object, therefore they show more interest towards the novel object (B). In order to compare the relative preference towards the novel object, a discrimination index (DI) was calculated as (B exploration time – A1 exploration time)/(B exploration time + A1 exploration time)^24^. A high DI value refers to the state when the animal sniffs longer at the novel object while a DI around 0 indicates the lack of novelty recognition.

Spatial learning and memory performance of the mice was measured using the Morris water maze test between the age of 2.5 to 5 months. A circular plastic pool (height: 35 cm, diameter: 105 cm) was filled with water (22 ± 1 °C). On day 0, a visible platform was placed into the pool with visual cues on the walls. Mice were allowed to swim for a maximum of 1 minute. From day 1, the escape platform (8-cm diameter) was submerged 1 cm below the surface of the water. Animals were trained for 11 consecutive days, with four trials per day. On the 5th and 10th day, the platform was removed, and the time and distance spent searching around a 24-cm diameter area over the original platform was calculated. On day 11, the hidden platform was re-positioned and the animals were let to swim four times for a maximum of 1 minute. Evaluation of behavioural tests was performed essentially as described in Gulyás *et al*.^26^.

### Cell cultures, transfection and chemical treatments

Primary cultures of embryonic hippocampal cells were prepared from C57Bl6/J (WT) or Caskin dKO mice on embryonic day 17–18, according to Czöndör *et al*.^27^. Cells were seeded onto poly-L-lysine-laminin (Sigma-Aldrich)–coated glass coverslips in 24-well plates at 1.45 × 10^5^ cells/well. Cells were cultivated for 13–16 day at 37 °C in 5% CO_2_, 95% air atmosphere. Transfection was carried out with Lipofectamine2000 (Invitrogen) using an empty backbone p-EGFP-N1 vector (Clontech) or a V5-tagged wild-type Caskin1 construct. The V5 epitope tagged Caskin1 was created by inserting the rat Caskin1 cDNA into a pcDNA3.1/TOPO-V5-His vector (V5-Caskin1)^18^. In order to detect transfected living neurons, we co-transfected an EGFP vector backbone together with the Caskin1-V5 construct using a 1:2 ratio of EGFP:Caskin1-V5 plasmids. Under these conditions, basically all V5 positive transfected neurons were also positive for the EGFP signal, allowing us to detect transfected neurons by EGFP positivity. Nevertheless, Caskin1-V5 overexpression was always verified in the investigated neurons by the anti-V5 immunocytochemistry (see below).

Chemically induced LTP (cLTP) was carried out at 37 °C. Cells were incubated in ECS buffer (150 mM NaCl, 2 mM CaCl_2_, 5 mM KCl, 10 mM HEPES, 30 mM glucose, 0.5 µM tetrodotoxin, 20 µM bicuculline and 1 µM strychnine; pH 7.4) for 5 min^28^. 200 µM glycine was applied for 3 min in ECS buffer, and then medium was changed back to the original medium, in which cells survived for an additional 5 h. In case of the APV-treated wells, APV was present in the buffers throughout the experiment. Chemicals and antagonists were all from Tocris Bioscience.

### Immunocytochemistry and microscopy in fixed cultures

Hippocampal cultures were fixed on the 13–16th day after plating for 20 min with cold 4% paraformaldehyde. Cultures were immunostained essentially as described in Czöndör *et al*.^27^. Primary antibodies were anti-Shank2 (guinea pig, 1:2000, Synaptic System), and anti-V5-tag (mouse, 1:1000, ThermoFisher Scientific). Appropriate biotinylated secondary antibody (anti-guinea pig, 1:1000, Jackson ImmunoResearch) was developed by Cy5-conjugated streptavidin (streptavidin-Cy5, 1:500, Jackson ImmunoResearch) while Alexa fluor-labeled secondary antibody was applied in 1:500 dilution (anti-mouse, 1:1000, Jackson ImmunoResearch). Images were taken with an LSM 800 (Carl Zeiss) or with a FluoView 500 LSM IX81 (Olympus) microscope with Plan Apochromat 63×/1.4 or Plan Apochromat 60×/1.4 immersion objectives. All experiments were repeated at least three times using independent cultures. Morphological characterization of dendritic protrusions was performed manually, as described by Bencsik *et al*.^24^.

### Co-immunoprecipitation and western blot

**Figure 1:**
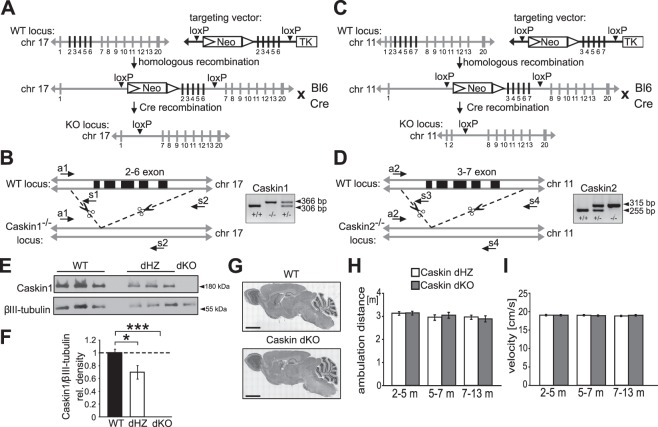
Generation of Caskin dKO mice. **(A**–**D**) Gene targeting strategies to knockout exons 2 to 6 of Caskin1 (**A**,**B**) or exons 3 to 7 of Caskin2 (**C**,**D**). In the targeting vectors, exons to be deleted were flanked by loxP sites. To achieve positive and negative selection, a neomycin (Neo) resistance gene cassette was inserted into intron 1 and the thymidine kinase gene (TK) was inserted downstream of exon 7 (Caskin1) or 8 (Caskin2), respectively. Mice carrying mutant floxed allele were crossed with transgenic C57Bl/6 (Bl6) mouse carrying Cre recombinase. **(B)** Position of deleted exons 2 to 6 are represented in chromosome 17. The primer set (a1, s1, s2) and the amplified regions (WT: 306 bp, KO: 366 bp) are marked on the wild type (WT) and KO locus. Right panel shows PCR genotyping of wild type (+/+), homozygous Caskin1 KO (−/−) and heterozygous (+/−) mice. **(D)** Position of deleted exons 3 to 7 are represented in chromosome 11. The primer set (a2, s3, s4) and the amplified regions (WT: 255 bp, KO: 315 bp) are marked on the wild type (WT) and KO sites. Right panel shows PCR genotyping of wild type (+/+), homozygous Caskin1 KO (−/−) and heterozygous (+/−) mice. **(E-F)** Comparison of endogenous Caskin1 levels between C57Bl6/J wild type (WT), heterozygous (dHZ) and Caskin dKO (dKO) mice at the age of 3 months. Quantitative data was based on 3 independent samples by normalizing Caskin1/βIII-tubulin ratio. **(G)** Median-sagittal sections of WT and dKO brains obtained at 3-months of age and stained by gallocyanine-chrome alum stains. Scale bar: 3 mm. **(H**–**I)** Total ambulation distance **(H)** and velocity **(I)** within the open field during aging. Caskin dHZ (n = 16; white columns) and Caskin dKO (n = 16; grey columns) data are displayed. All data are shown as mean ± SEM. *p < 0.05; ***p < 0.001.

Mouse brain tissues from C57Bl6/J (WT), Caskin dHZ and Caskin dKO mice were homogenized in ice-cold lysis buffer (1% Triton X-100, 100 mM NaCl, 30 mM Tris pH 7.5, 1 mM EGTA, 10 mM NaF, 1 mM Na_3_VO_4_, 2 mM *p*-nitrophenyl-phosphate, 10 mM benzamidin, 1 mM phenylmethylsulphonyl fluoride, 25 μg/ml each of aprotinin, pepstatin A and trypsin inhibitor) by suspension with a cut-end pipette tip. The homogenates were centrifuged first at 4000 rpm for 2 min then 14000 rpm for 10 min in 4 °C to remove cell debris. Thereafter, 25 µL of BSA-blocked Protein A (Sigma-Aldrich) beads were added, and the samples were incubated for 1 h at 4 °C. Lysates were incubated with specific antibodies (anti-Caskin1, rabbit, 1:166, Synaptic Systems; anti-Shank2, guinea-pig, 1:166, Synaptic System) for 1 hour on ice. Immunoprecipitates were washed three times with ice-cold PBS, containing 0.4% Triton X-100 (Sigma-Aldrich). Brain lysates and precipitated complexes were separated by SDS-PAGE, transferred to nitrocellulose or PVDF (polyvinylidene difluoride, Merck) membrane. After blocking with 0.5% blocking reagent (Roche Diagnostics) in TBST (Tris-buffer containing 0.05% Tween-20 and 0.1% NaN_3_), membranes were probed with specific antibodies as follows: anti-Caskin1 (rabbit, 1:1000, Synaptic Systems), anti-GluA1 (mouse, 1:000, Merck), anti-phospho-GluA1 (pS831) (rabbit, 1:1000, Merck), anti-phospho-GluA1 (pS845) (rabbit, 1:1000, AbCam), anti-Shank2 (guinea-pig, 1:2000, Synaptic System), anti-PSD-95 (mouse, 1:1000, ThermoFisher), anti-Caskin2 (rabbit, 1:1000, AbCam), anti-Caskin2 (rabbit, 1:1000, ThermoFisher), anti-Caskin2 (mouse, 1:1000, Santa Cruz) and anti-βIII-tubulin (mouse, 1:5000, Exbio). Signals were visualized with horseradish peroxidase-coupled secondary antibodies (1:20000; Dianova) using enhanced chemiluminescence (ECL) detection reagents (Amersham Pharmacia Biotech). Average intensity values were calculated using Image Studio Lite 5.0 software as specific signals/βIII-tubulin signals and were normalized to the corresponding control values. Complete images of the Western blot cut-outs presented in Figures 1E and 7B–H are displayed in Suppl. Figures S1, S5 and S6.

### Electronmicroscopy

For immunoperoxidase staining, hippocampal cultures were fixed on the 11–12th day after plating, followed by 4% paraformaldehyde, 0.5% glutaraldehyde and 0.2% picric acid in 0.1 M PB (pH 7.4). The sections were incubated in 2% NGS for 60 min at room temperature to suppress non-specific binding. Sections were incubated in anti-V5 IgG (mouse, 1:1000, Invitrogen) for 1 day at 4 °C. After thorough washing with PBS, sections were incubated with anti-mouse biotin (1:500, Vector) in blocking solution for 1 day and then in avidin-peroxidase for 1 h; immunopositive structures were visualized using Ni-DAB.

2–5 months old Caskin dHZ and Caskin dKO male mice were deeply anesthetized with chloral-hydrate (350 mg/kg, i.p.), then perfused intracardially with saline, followed by 4% paraformaldehyde, 0.5% glutaraldehyde and 0.2% picric acid in 0.1 M PB (pH 7.4). 50 µm coronal sections were cut with a Vibratome (Leica). Plastic-embedded ultrathin sections were examined in a JEM1011 electron microscope (JEOL) at 60 kV and images were taken with an Olympus Morada 11 megapixel camera and iTEM software (Olympus). Electron micrographs of randomly selected fields were taken from hippocampus CA1 stratum radiatum. Length of the active zone, synaptic area, PSD length and profile circularity (4π × area/perimeter^2^) were measured using NIH ImageJ. For every clearly-defined synapse, the lateral edges of the postsynaptic density were defined.

### Electrophysiological recordings

Miniature excitatory postsynaptic currents (mEPSC) experiments were performed essentially as described in Szíber *et al*.^29^. Electrophysiological recordings of dissociated primary hippocampal neurons (DIV14–17) were performed under an A1 Axiovert 200 M microscope (Carl Zeiss). mEPSCs were recorded at room temperature (21–23 °C) under whole cell conditions. Signals were amplified using a MultiClamp700B (Molecular Devices) and acquired at 20 kHz using the data acquisition software DASYLab version 11 (National Instruments). The composition of the bath solution (ACSF) was (in mm): NaCl 140, KCl 5, CaCl_2_ 2, MgCl_2_ 1, HEPES 5 and glucose 10, pH 7.4. 500 nM TTX was used to block spike mediated neurotransmission. Patch electrodes were filled with the following solution (in mM): K-gluconate 100, KCl 10, KOH 20, MgCl_2_ 2, NaCl 2, HEPES 10, EGTA 0.2 and glucose 5; pH 7.3, yielding an electrode resistance of 5–8 MΩ. Membrane potential in voltage clamp experiments was held at −60 mV. Analysis of mEPSC frequency and amplitude was performed semi-automatically by a software developed by A. Szűcs (NeuroExpress). Time constants of the miniature EPSCs were determined by fitting the decay parts of individual events with monoexponential functions using the Levenberg-Marquard algorithm. Events with amplitude over 10 pA were selected for the nonlinear fitting and the gradual decrease of the χ^2^ goodness-of-fit parameter was evaluated to accept events as successfully fitted.

Hippocampal LTP experiments were performed essentially as described in Borbély *et al*.^30^. Caskin dHZ and Caskin dKO were decapitated under chloral-hydrate (350 mg/kg, i.p.) anesthesia, and 400-µm-thick horizontal hippocampal slices were cut. After 1 h regeneration in the incubation solution containing HEPES-buffer, slices were transferred to a Haas-type recording chamber (Experimetria Ltd.) through which standard artificial cerebrospinal fluid (ACSF) was perfused using a peristaltic pump. The solution was saturated with carbogene (95% O_2_ and 5% CO_2_). Glass microelectrodes filled with 1 M NaCl (3–10 MΩ resistance) were positioned as recording electrodes in the stratum pyramidale of the hippocampal CA1 region. Slices were tetanized with the intensity that elicited 70% of maximal population spike (POPS) amplitude at the Schaffer-collaterals. At the beginning of the data recording session, 0.1 Hz triggers were applied for 10 min (test stimulation), and then HFS (high frequency stimulation) trains (100 Hz, 5 sec) were applied for LTP induction. Averaged responses were calculated as a mean of amplitude of six POPSs before and following LTP induction.

E/S curves displaying the ratio between the derivative of the excitatory postsynaptic potential (dEPSP) and the POPS amplitude were determined. According to Wheal *et al*.^31^; a left or a right shift of the E/S curve indicates facilitation or depression, respectively. HFS-dependent changes were visualized by vectors originating from the pre-HFS and pointing to the post-HFS values of the same recordings as a function of POPS amplitude change and dEPSP values. To compare the direction of these vectors, the four-quadrant inverse tangent method^32^ was used to create polar histograms, where 0° and 90° determine the direction of the x- and y-axis (thus, dEPSP and POPS amplitude values), respectively.

In order to compare the relative E/S facilitation/depression changes of dHZ and dKO recordings, individual pre- and post-HFS dEPSP and POPS amplitude values were normalized to the peak of the corresponding pre-HFS values of the same experimental group. The normalized values were plotted and the orthogonal distance from the x → y = x function was calculated as an algebraic value (see dotted distances from the dashed diagonal on Fig. 3H). When the average distance value is positive, it indicates facilitation (left shift from diagonal), while a negative distance value represents depression (right shift from diagonal).

### Statistical analyses

Statistical analysis was performed using IBM SPSS Statistics v.21.0.0.0 (SPSS Inc., Chicago, USA). Student’s *t-test* or non-parametric Mann-Whitney test was used for two-group comparative analyses. For multiple-group comparisons Tukey *post-hoc* was applied. Statistical significance was declared if the *p* values were under 0.05. Data are displayed as mean ± SEM, unless otherwise indicated.

## Results

### Deletion of Caskin1 and Caskin2 does not lead to severe behavioural changes

Caskin1 and 2 knockout mice were generated by targeted disruption of *Caskin1* gene on chromosome 17 (Caskin1^−/−^ mice) or *Caskin2* gene on chromosome 11 (Caskin2^−/−^ mice), respectively (Fig. 1A–D). Successful deletion of exon 2 to 6 (*Caskin1* gene) or exon 3 to 7 (*Caskin2* gene) was demonstrated by PCR analyses (Fig. 1B,D). Double knockout Caskin1^−/−^/Caskin2^−/−^ mice (designated as Caskin dKO animals) were generated by breeding heterozygous Caskin1^+/−^ and Caskin2^+/−^ mice and genotyping the offsprings. Western blot analyses from brain lysates of C57Bl6/J wild-type (Caskin1^+/+^/Caskin2^+/+^, indicated as WT), double heterozygous (Caskin1^+/−^/Caskin2^+/−^; designated as Caskin dHZ) and Caskin dKO mice revealed that endogenous Caskin1 expression normalized to neuron-specific βIII-tubulin values is reduced in Caskin dHZ compared to Caskin dWT and is non-detectable in Caskin dKO animals (Fig. 1E,F). Unfortunately, neither of the tested Caskin2-specific antibodies gave reliable Western blot signals (Supplementary Fig. S1). Although in this study we wished to focus on Caskin1 protein, it was decided to knockout both Caskin proteins to avoid the potential compensatory effect of Caskin2 in the Caskin1 knockout mice. Breeding differences between Caskin dWT, dHZ and dKO mice were not observed (data not shown). Basic brain anatomy was evaluated by gallocyanine-chrome alum staining. Images did not reveal large-scale differences between the investigated brains (Fig. 1G). No motor coordination defects were detected in the open field test during the development of Caskin dHZ and dKO mice, as ambulation distance and average velocity of the mice were similar during the 5-minute observation time in young (2–5 months of age), middle aged (5–7 months old) or aged (7–13 months old) mice (Fig. 1H,I).

### Lack of endogenous Caskin1 and Caskin2 impairs novel object recognition and spatial memory

**Figure 2:**
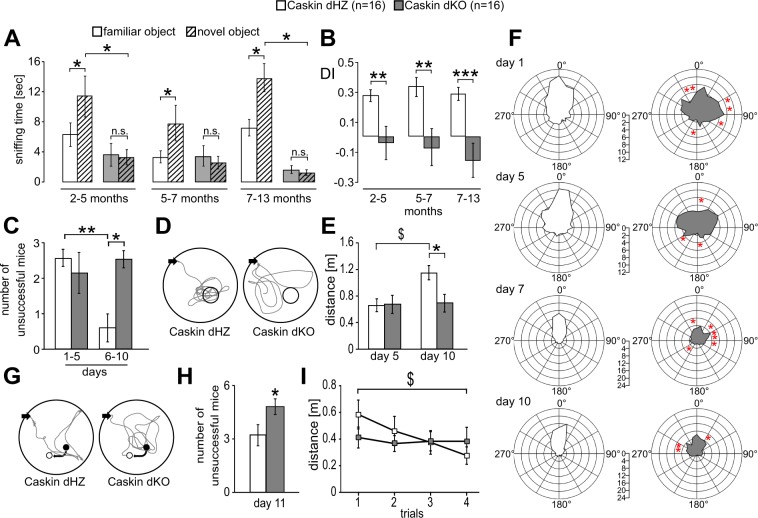
Deletion of Caskin1 and Caskin2 genes impairs novel object recognition and spatial memory when comparing Caskin double heterozygous (Caskin dHZ, designated by white colour; n = 16) and Caskin double knockout (Caskin dKO, designated by grey colour; n = 16) mice. **(A**,**B)** Novel object recognition tests performed at different developmental ages, indicating **(A)** the average sniffing time what Caskin dHZ (columns with white background) and dKO (columns with grey background) mice spent at the familiar (A1; empty columns) or novel (B; striped columns) objects in the 2^nd^ round of novel object recognition test, performed after 5 hours. **(B)** Discrimination index showing the relative preference towards the novel object (DI; see Materials and Methods) was significantly lower in Caskin dKO mice, regardless of their developmental age. **(C–I)** Morris water maze test results of Caskin dHZ and Caskin dKO mice. **(C)** Average number of failures during day 1–5 and 6–10. **(D)** Representative trajectories from Caskin dHZ and Caskin dKO mice during the second probe trial of the Morris water maze (day 10). Black arrows indicate start positions; circles represent the target area over the original platform. **(E)** Average swimming path length above the removed platform during the probe trials on day 5 and 10. **(F)** Average angle preference values of Caskin dHZ and Caskin dKO mice obtained on day 1, 5, 7 and 10. **(G**–**I)** On day 11, the platform was moved from its original position (filled circle) into an adjacent quadrant (open circle) **(G)**. Black arrows indicate the start position of the swimming trajectories. **(H)** Average number of failures to find the relocated platform. **(I)** Average swimming path length during four consecutive trials on day 11. All data are shown as mean ± SEM. Asterisks represents significance compared to control values (white columns), $ indicates significant differences between data pairs. ^$^p < 0.05; *p < 0.05; **p < 0.01; ***p < 0.001.

In order to obtain a more specific insight whether deletion of endogenous Caskin proteins influence learning ability, we compared novelty recognition as well as spatial navigation skills between Caskin dHZ and dKO male animals (Fig. 2). The hippocampus-independent novel object recognition test was carried out as described in the Materials and Methods section. As displayed on Fig. 2A, all tested Caskin dHZ animals sniffed the novel object significantly more than the familiar object (compare white columns with and without stripes), resulting in a discrimination index around 0.3 (Fig. 2B). On the other hand, Caskin dKO mice sniffed at the novel and the familiar objects for the same time (compare grey columns with and without stripes on Fig. 2A), resulting in a significantly reduced discrimination index around or below 0 (Fig. 2B). Thus, Caskin dKO animals were unable to discriminate between the familiar and novel objects, regardless of their age.

Hippocampus-dependent spatial memory and re-learning abilities were examined in the Morris water maze, with 2.5 to 5 months old Caskin dHZ and dKO animals (Fig. 2C–I). Animals were tested for 11 consecutive days, with 4 swimming sessions daily. Average swimming speed remained the same and did not differ between Caskin dHZ and dKO animals (17.03 ± 0.32 and 16.34 ± 0.32 cm/s, respectively). Caskin dHZ mice efficiently learnt the position of the hidden platform, as the number of animals failing to reach the platform within the 1-minute-long sessions was gradually reduced during the trainings (see Suppl. Fig. S3 for the daily records). Caskin dKO mice, on the other hand, had similar failure rates during the initial or later phases of trainings. When the average number of unsuccessful animals was calculated between day 1–5 and 6–10, dHZ mice performed significantly better during day 6 to 10 (Fig. 2C).

During the teaching sessions, the direction of swimming path was evaluated within a 360° plot (Fig. 2F), where orientation was calculated as the degree between the actual direction of swim and the location of the platform. Caskin dHZ mice performed platform-oriented swim already from day 7 on, while Caskin dKO animals had significantly less preference to swim directly towards the hidden platform even on day 10.

During additional swimming tests on day 5 and 10 (probe trials), the hidden platform was removed and the distance swam above the close vicinity of the removed platform was evaluated (Fig. 2D–E). On day 5, the performance of Caskin dHZ and dKO mice did not differ significantly. 5 days later, Caskin dHZ mice improved their performance as the swimming path above the removed platform was significantly increased, indicating a more efficient spatial learning compared to Caskin dKO animals. Taken together, lack of Caskin proteins led to impaired hippocampal spatial learning abilities.

On day 11, the hidden platform was re-positioned (Fig. 2G) and the animals were let to swim four times for a maximum of 1 minute with 30 minutes intervals (Fig. 2H–I). Caskin dHZ mice were significantly more efficient in finding the re-located platform (Fig. 2H) and the swimming distance needed to reach the platform was significantly improved during the trials compared to Caskin dKO mice (Fig. 2I). These data indicate that Caskin dKO mice have less ability to modify already formed spatial memories.

### Endogenous caskin regulates LTP formation in acute hippocampal slices

**Figure 3:**
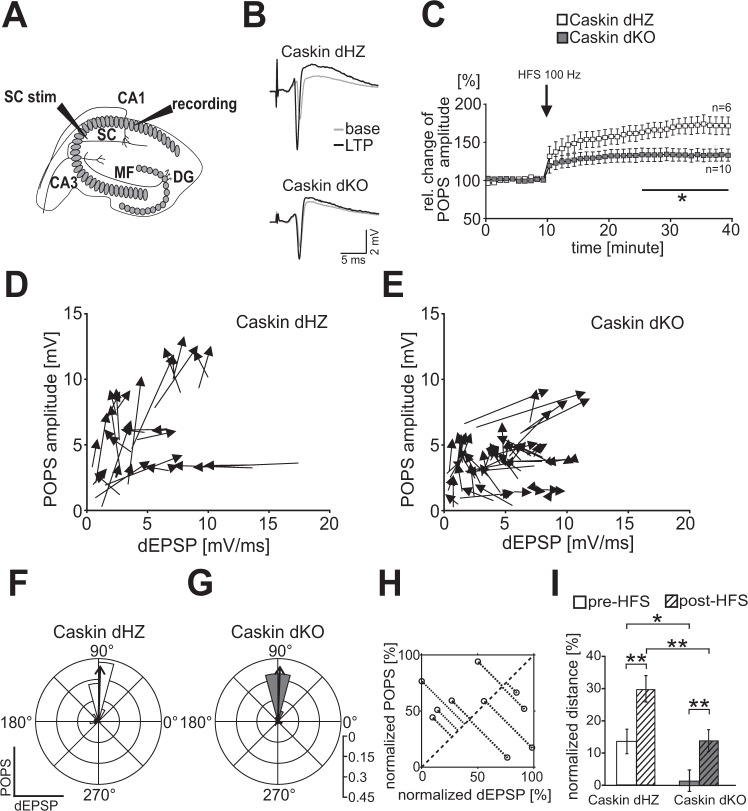
LTP formation is impaired in Caskin dKO hippocampal slices. **(A)** Stimulation paradigm during LTP induction. (**B**) Representative sample traces recorded before (grey) and after (black) the high-frequency stimulation in the CA1 str. pyramidale in Caskin dHZ and Caskin dKO mice. **(C)** Relative change of POPS amplitudes after the application of 100 Hz stimulus for 5 sec (HFS) to the Schaffer collaterals (SC stim). Asterisk represents significant differences compared to Caskin dHZ values. *p < 0.05. **(D,E)** Vectors displaying the transition from the pre-HFS to the post-HFS state in dHZ (**D**) and dKO (**E**) recordings are plotted as a function of the derivative of the excitatory postsynaptic potential (dEPSP) and the POPS amplitudes (POPS). **(F, G)** 15° polar histograms show the distribution of pre-HFS → post-HFS vector directions calculated from Caskin dHZ **(F)** and Caskin dKO **(G)** recordings. The thick arrow within the histogram indicates the median of vector directions. **(H**) Cartoon depicting the determination of orthogonal distance for pre-HFS and post-HFS data point. Dotted lines demonstrate the orthogonal distance from the x → y = x function (indicated by the dashed diagonal). Graph shows randomized data. **(I)** Comparison of normalized orthogonal distance values before or following HFS in Caskin dHZ and Caskin dKO mice. *p < 0.05; **p < 0.01. All data are shown as mean ± SEM. See Materials and Methods for a detailed description of data evaluation.

As Caskin dKO mice exhibited impaired memory formation, we elucidated the consequences of deleting Caskin1 and 2 on hippocampal long-term potentiation (LTP; Fig. 3A–I). Acute hippocampal slices obtained from 5–7 months old Caskin dHZ and dKO mice were stimulated by 100 Hz (HFS) via the Schaffer-collaterals and field potentials were recorded from the CA1 str. pyramidale (Fig. 3A, sample traces before and after HFS are shown in Fig. 3B). Before HFS, average POP-spike amplitude was 4.18 ± 0.75 mV and 3.15 ± 0.33 mV in Caskin dHZ and dKO animals, respectively, indicating that the basal excitability did not differ significantly among the investigated groups (p = 0.173; Student’s t-test). In case of Caskin dHZ slices, LTP developed relatively slowly and reached a 73.8 ± 11% relative increase in population spike (POPS) amplitudes by 30 minutes following HFS. On the other hand, relative POPS amplitude was elevated only by 33.2 ± 8% and remained stable in Caskin dKO slices (Fig. 3C). Thus, the extent of LTP formation in Caskin dKO slices was significantly reduced from 15 minutes following HFS.

In order to have a more detailed evaluation of the observed differences in POPS amplitudes after HFS stimulus, E/S curves displaying the ratio between the derivative of the excitatory postsynaptic potential (dEPSP) and the POPS amplitude were determined^31^. The differences between the baseline and the post-tetanic stimulation values were visualized by vectors originating from the pre-HFS and pointing to the post-HFS values of the same recordings (Fig. 3D,E). The range of pre-HFS → post-HFS vector directions was compared using polar histograms (see Material and Methods; the median of the vector directions is indicated by a thick arrow in Fig. 3F,G). As in both cases, pre-HFS → post-HFS vectors were similarly oriented towards 90° (p = 0.22, Kolmogorov-Smirnov test), our analysis indicates that mild or no dEPSP rise was accompanied by a robust POPS amplitude increase upon tetanic stimulation in both groups. Thus, HFS induces facilitation in Caskin dHZ and dKO hippocampal slices.

To compare the extent of facilitation between the different genotypes, normalized orthogonal distances of the pre- and post-HFS data points were calculated from a normalized dEPSP/POPS amplitude plot (see Fig. 3H and a detailed description of the analysis within the Materials and Methods section). As post-HFS normalized distance values were increased compared to the pre-HFS values in both Caskin dHZ and Caskin dKO recordings, our results indicate that E/S facilitation was induced by HFS in both groups but the baseline and the post-HFS values were significantly lower in the Caskin dKO group (Fig. 3I).

These results implicate that Caskin deficiency reduces synaptic efficacy under normal conditions and during LTP formation in hippocampal slices but does not block the capability for facilitation. Our detailed analysis also suggests that the increase in synaptic efficacy is mainly due to post-synaptic effects.

### Caskin proteins regulate dendritic spine morphology in CA1 str. radiatum *in vivo*

**Figure 4:**
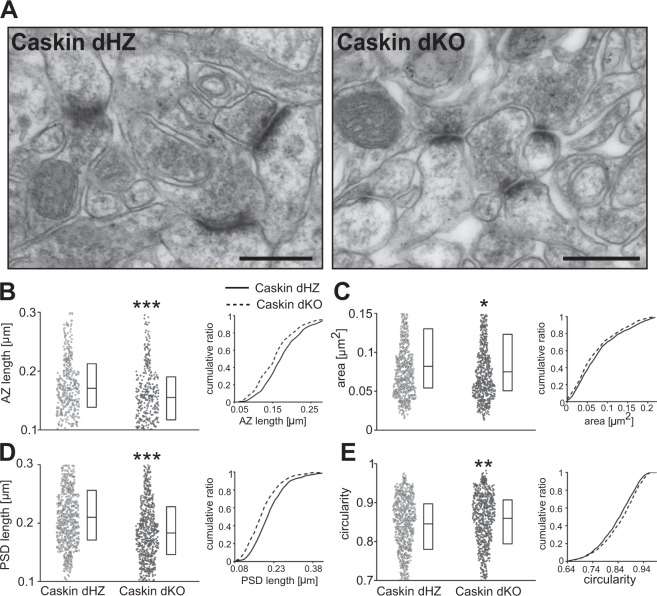
Ultrastructural analysis in the hippocampal CA1 str. radiatum reveals that the lack of Caskin1 and Caskin2 leads to smaller dendritic spines. **(A)** Representative electron micrographs from the CA1 region. Scale bars indicate 300 nm. **(B–E)** Quantitative evaluation of dendritic spine morphology of Caskin dHZ (n = 561; 3 mice, light grey) and Caskin dKO (n = 552; 3 mice; dark grey) littermates, displaying average active zone length **(B)**, spine area **(C)**, PSD length **(D)** and circularity of the dendritic spines **(E)**. Boxplots show the median (middle lines) and the 25–75 percentiles of data while individual dots represent single spine values. Cumulative distribution functions are displayed on the right, with solid and dashed lines representing Caskin dHZ and Caskin dKO data, respectively. Asterisks represent significant differences compared to Caskin dHZ values. *p < 0.05; **p < 0.01; ***p < 0.001.

Impaired hippocampal LTP formation can be due to alterations within excitatory synapses. To our knowledge, there are no commercially available antibodies against Caskin1 or 2 which reliably detect endogenous proteins in light- or electronmicroscopy. Thus, we were restricted to perform ultrastructural analyses in Caskin dHZ and dKO hippocampus, concentrating on the organization of excitatory synapses within the str. radiatum of CA1 pyramidal neurons (Fig. 4A–E). Over 1000 synapse profiles were analysed from 3–3 Caskin dHZ and dKO mice. Our analysis revealed that active zone length, spine area and the length of the postsynaptic density within the spine heads were significantly reduced, while spines possessed a rounder shape in Caskin dKO CA1 neurons (Fig. 4B–E). These results indicate that the lack of Caskin proteins leads to morphological alteration of both pre- and postsynaptic profiles within the hippocampus.

### Caskin proteins do not influence synaptic release or postsynaptic mEPSCs

Ultrastructural analysis indicated altered synaptic organization in Caskin dKO hippocampus. Whole-cell patch clamp recordings can provide useful information on synaptic transmission and can determine whether pre- or postsynaptic alterations are the primary cause for the observed reduction in hippocampal LTP formation in acute slices. Unfortunately, technical difficulties hindered patch clamp measurements in hippocampal acute slices. Therefore, we performed whole-cell voltage-clamp recordings in dissociated hippocampal neuronal cultures – although these experiments can not provide a direct explanation for the observed alterations in the synaptic profiles *in vivo*, they can reveal whether spontaneous synaptic transmission is selectively altered by the lack of Caskin proteins.

Whole-cell voltage clamp recordings were carried out in DIV14–17 Caskin WT, dHZ and dKO dissociated neuronal cultures. Spike-mediated synaptic transmission was blocked by 500 nM TTX and interevent interval (IEI) times, mEPSC amplitudes as well as mEPSC decay times were determined (Suppl. Fig. 3). While the frequency of mEPSC events primarily depends on the presynaptic release probability, mEPSC amplitude is determined by the neurotransmitter content of the synaptic vesicles, the density and/or the conductance of postsynaptic neurotransmitter receptors. Somewhat surprisingly, none of the analysed parameters differed significantly between WT, Caskin dHZ and dKO neurons under normal conditions (Suppl. Fig. 3A,B).

We also tested whether the overexpression of Caskin1 protein has any electrophysiological consequences *in vitro*. Dissociated cultures were transfected with a V5 epitope-tagged wild-type Caskin1 construct in combination with the EGFP vector backbone or with the EGFP vector itself as control. 24 hours later, transfected neurons were identified by their fluorescence and were subjected to whole cell patch clamp measurement. Overexpression of Caskin1 in the recorded cells was verified following fixation and anti-V5 immunostaining (data not shown). As presented in Suppl. Fig. 3C,D, overexpression of Caskin1 did not have any significant effects on mEPSCs characteristics.

Taken together, these data indicate that Caskin proteins do not have a detectable effect on synaptic release or postsynaptic current formation under activity deprived conditions *in vitro*, neither in Caskin dHZ or dKO cultures nor upon overexpressing Caskin1 in Caskin dKO neurons.

### Caskin1 regulates protrusion density and morphology in cultured hippocampal neurons

**Figure 5:**
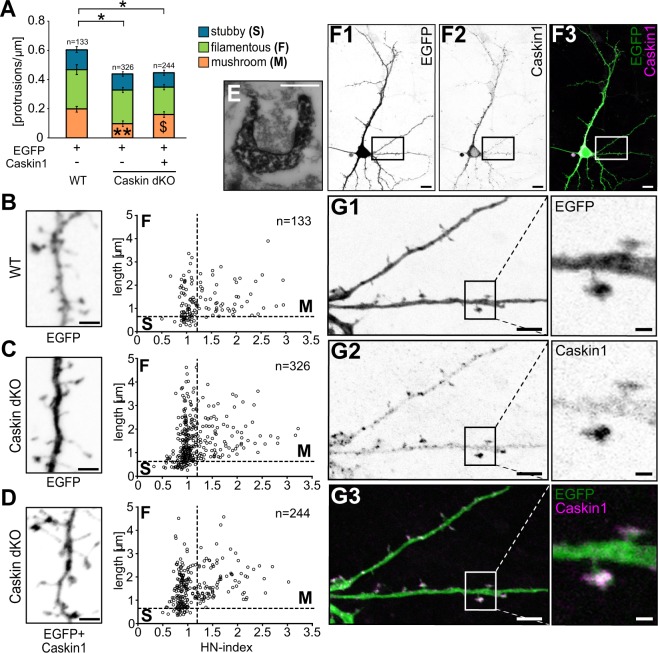
Caskin levels influence protrusion density and dendritic spine morphology in cultured hippocampal neurons. **(A–D)** Morphological characteristics of dendritic protrusions in EGFP-transfected C57Bl6/J (WT) neurons or Caskin dKO neurons overexpressing EGFP only or EGFP in combination with V5-tagged Caskin1. **(A)** Mean protrusion density on the tertiary dendritic branches, based on the morphological classification of protrusions as stubby, filamentous or mushroom^24^. Data were obtained from three independent cultures; the number of spines is indicated in the figures. All data are displayed as mean ± SEM. Asterisks represent significance compared to C57Bl6/J (WT) EGFP-expressing values and the $ symbol indicates significant differences between protrusion categories. *p < 0.05; **p < 0.01; ^$^p < 0.05. **(B–D)** Dendritic protrusion morphology in EGFP-transfected C57Bl6/J WT neurons (**B**), Caskin dKO neurons overexpressing EGFP **(C)** and Caskin dKO neurons transfected with EGFP and V5-tagged Caskin1 constructs **(D)**. Dots represent individual protrusions, plotted along the head/neck width ratio (HN-index) and length. Representative inverted fluorescent images of the EGFP signal are shown on the left side. Scale bar: 2 µm. **(E)** Electron microscopy detection of anti-V5 immunoreactivity by DAB precipitates in a dendritic spine of a CD1 neuron overexpressing V5-tagged Caskin1 protein. Scale bar: 200 nm. (**F**–**G)** Inverted fluorescent and merged images of a CD1 hippocampal neuron, expressing EGFP and V5-tagged Caskin1. Boxed areas on **(F**,**G)** designate the enlarged ROIs on **(G)**. Scale bars indicate 10 µm **(F1-3)**, 5 µm (left figures in **G1-3**) or 1 µm (enlarged ROIs on the right in **G1-3**).

Synaptic plasticity is known to regulate the size and number of dendritic spines in an activity-dependent manner^33^. In order to investigate Caskin1-mediated effects on dendritic spine formation and morphology, dissociated embryonic hippocampal primary neuronal cultures were prepared from C57Bl6/J wild-type (WT) and Caskin dKO brains. Similarly to whole-cell patch clamp recordings, Caskin dKO cultures were transfected with a V5 epitope-tagged wild-type Caskin1 construct in combination with the EGFP vector backbone or with the EGFP vector itself as control. Transfected neurons were analysed 24 hours later, by anti-V5 immunostaining (Fig. 5A–D).

Average protrusion density in Caskin dKO neurons transfected only with EGFP was significantly reduced compared to EGFP-expressing WT neurons but was not influenced by Caskin1 overexpression (Fig. 5A). On the other hand, when morphological parameters of the protrusions were analysed in more detail (see Fig. 5B–D, plotting the length along the head-to-neck ratio [HN-index] of the individual protrusions^24^); the density of mushroom-like protrusions in Caskin dKO neurons was significantly lower than in wild-type neurons. Overexpression of Caskin1 in Caskin dKO neurons increased significantly the ratio of mushroom-like protrusions in expense for thin, filopodial-like protrusions (Fig. 5A).

Overexpressed Caskin1 was detected predominantly within the somatodendritic region of the transfected neurons, with local enrichment within the dendritic spine heads (Fig. 5F,G). Ultrastructural analyses in Caskin1-V5 and EGFP cotransfected neurons were processed by anti-V5 pre-embedding immunocytochemistry and visualized by DAB precipitates. Our results proved that overexpressed Caskin1 was indeed present in postsynaptic dendritic spines forming synapses (see Fig. 5E).

Taken together, our data indicate that lack of endogenous Caskin proteins leads to reduced PSD length and shrinkage of dendritic spines *in vivo* and decreased protrusion density *in vitro*, while overexpression of Caskin1 leads to the expansion of dendritic spine heads in cultivated neurons within 24 hours following transfection.

### Lack of endogenous Caskins does not affect cLTP-induced morphological changes in the protrusions of cultured hippocampal neurons

**Figure 6:**
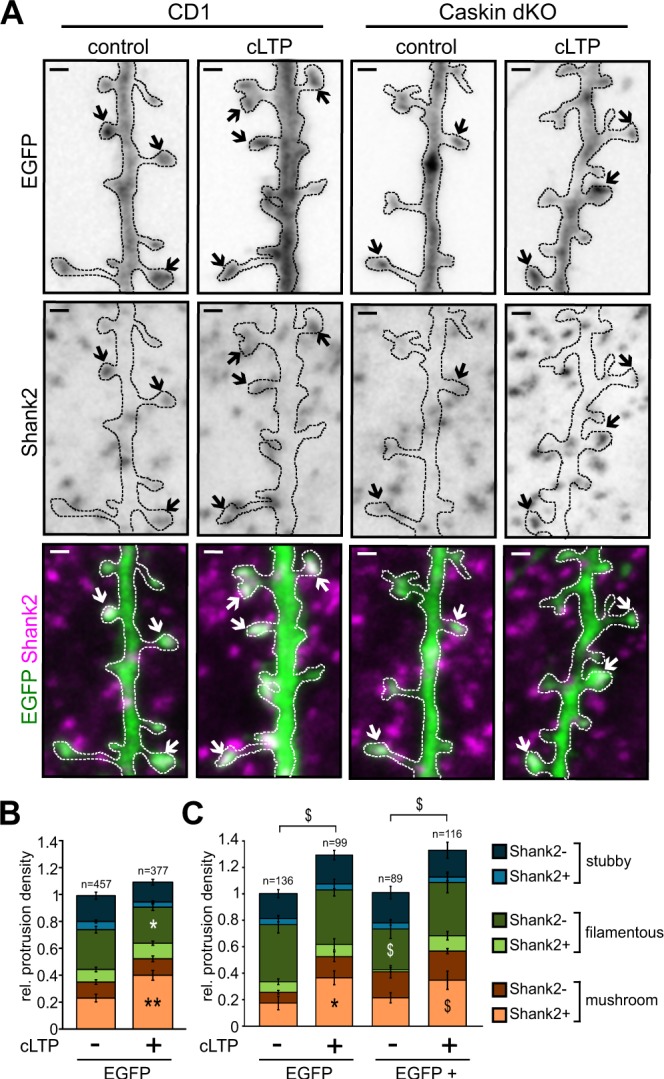
Caskin1 does not influence cLTP-induced morphological changes in hippocampal neuronal cultures. **(A)** Representative images of CD1 and Caskin dKO neurons under control conditions or 5 hours after a 3-min induction by glycine treatment (cLTP). Arrows indicate mushroom spines of EGFP expressing dendritic branches, stained with anti-Shank2. Bars: 1 µm. **(B**,**C)** Mean relative protrusion density on the tertiary branches of EGFP-expressing CD1 **(B)** or Caskin dKO **(C)** hippocampal neurons with or without cLTP treatment. Protrusion types are categorized according to their morphology (stubby, filamentous or mushroom) and Shank2 positivity. **(C)** Caskin dKO neurons were transfected with either EGFP only or EGFP and V5-tagged Caskin1 constructs, 24 hours before the cLTP treatment. Data were obtained from 3–4 independent cultures. All data are displayed as mean ± SEM. Asterisks represent significance compared to control cultures and the $ symbol indicates significant differences between protrusion categories. *p < 0.05; **p < 0.01, ^$^p < 0.05. The number of analysed protrusions is indicated above the columns.

Protrusion density in Caskin dKO hippocampal neurons was reduced in comparison to C57Bl6/J wild-type cultures (see Fig. 5A). In order to test whether the presence or absence of Caskin proteins influence the ratio of transient or stabilized connections, Shank2 immunostaining was combined with the morphological analysis of dendritic protrusions (Fig. 6). Protrusions were classified according to their morphological features based upon their length and HN-index (see Fig. 5B–D). Stubby, filamentous and mushroom-like protrusions were then subdivided according to the presence or absence of Shank2 immunopositivity. In this way, we aimed to separate stabilized spines containing PSD from those transient filopodia which did not form functional synapses yet (Fig. 6A–C and Suppl. Fig. S4). For technical reasons, CD1 wild-type (Fig. 6A,B) and Caskin dKO (Fig. 6A,C) hippocampal cultures were both used in these studies. Importantly, comparison of EGFP-expressing wild-type neurons obtained from CD1 and C57Bl6/J embryos revealed that when these dissociated neurons are maintained under identical culture conditions, protrusion density and morphology are very similar despite the different mouse strains used (Suppl. Fig. S4). Thus, dendritic protrusion density and Shank2 positivity between EGFP expressing Caskin dKO and CD1 wild-type cultures can be compared.

Our investigations revealed that the lack of Caskin proteins significantly increased the ratio of Shank2 negative, filamentous protrusions in Caskin dKO neurons compared to CD1 cultures (0.2989 ± 0.02 versus 0.4309 ± 0.05, respectively; p = 0.025; compare dark green columns in Fig. 6B,C). This effect was reverted within 24 hours, when V5-tagged Caskin1 was overexpressed in Caskin dKO neurons (Fig. 6C; 0.3082 ± 0.06, p = 0.0301). The ratio of Shank2 positive or negative stubby and mushroom-like protrusions, on the other hand (see light and dark columns coloured blue and orange, respectively), did not differ significantly (Fig. 6B,C).

It is well-known that increased synaptic efficacy during LTP is accompanied by long-lasting structural changes, including the expansion of dendritic spine heads^34,35^. To test whether Caskin proteins have any role during LTP-dependent structural changes, we evoked glycine-induced chemical LTP (cLTP) in CD1 and Caskin dKO cultures, transfected with the EGFP vector backbone alone or in combination with the V5-tagged Caskin1 and performed the above mentioned protrusion classification (Fig. 6A–C).

In agreement with previously reported data^28,36^, cLTP treatment significantly increased the ratio of Shank2-positive mushroom spines (indicated with light orange) and decreased the amount of Shank2-negative filopodia (see dark green columns) in CD1 neurons (Fig. 6B). Overall protrusion density was slightly but not significantly elevated 5 hours following the onset of the 3-minute long glycine treatment. Caskin dKO neurons, on the other hand, responded to cLTP with a significant increase in protrusion density and an elevation in the ratio of Shank2-positive mushroom spines (light orange columns in Fig. 6C). Importantly, overexpression of V5-tagged Caskin1 protein in Caskin dKO neurons did not affect cLTP-induced changes (Fig. 6C). These data indicate that the cLTP-evoked morphological redistribution of dendritic protrusion types is not affected by altered Caskin 1 or 2 levels. Interestingly, both the deletion of Caskin proteins or the overexpression of Caskin1 enhance cLTP-dependent increase in protrusion density.

### Caskin1 co-localizes and precipitates with endogenous Shank2, the master scaffold of the postsynaptic density

**Figure 7:**
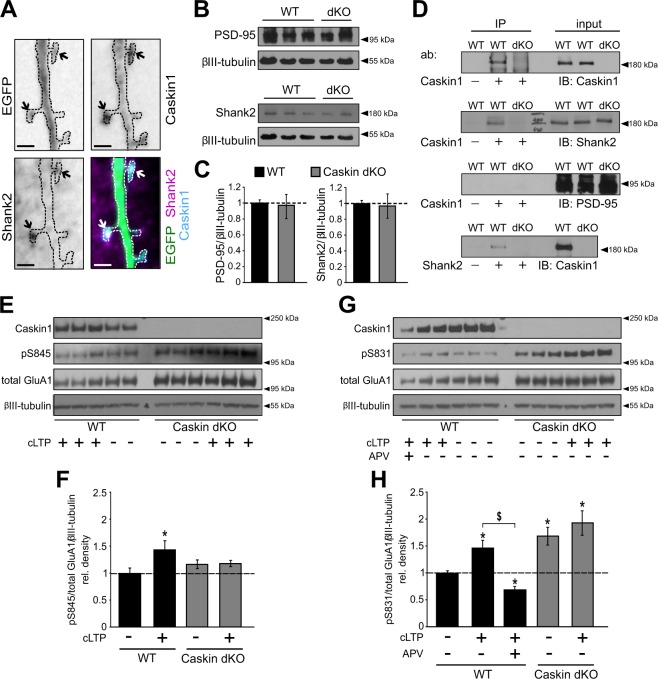
Caskin1 co-localizes with the postsynaptic marker Shank2 protein and regulate the phosphorylation of GluA1 AMPA receptor subunit. **(A)** Representative images of a Caskin dKO hippocampal neuron overexpressing EGFP and V5-tagged Caskin1 and immunostained for the presence of endogenous Shank2 protein. Arrows indicate mushroom spines. Bars indicate 2 µm. **(B,C**) Comparison of endogenous PSD-95 and Shank2 levels in C57Bl6/J wild type (WT) and Caskin dKO brain lysates at the age of 3 months. (**C**) PSD-95 and Shank2 levels were normalized to the corresponding βIII-tubulin intensity values. In case of independent western blots, Caskin dKO ratios were normalised to WT values on the same blot and then normalised values were averaged between the independent Western blots. **(D)** Brain lysates of wild-type (WT) and Caskin dKO mice were immunoprecipitated with anti-Caskin1 or anti-Shank2 antibodies, and detected by antibodies against Caskin1, Shank2 and PSD-95 (IB). **(E**–**H)** Detection and quantification of S845 and S831 phosphorylation and total GluA1 levels in DIV14-16 hippocampal neuronal cultures isolated from wild-type (WT) or Caskin dKO embryos, with our without cLTP treatment. To block NMDA-receptor dependent changes, 50 μM APV was also applied. βIII-tubulin was detected as a loading control. Graphs were calculated from 4–5 independent cultures by normalizing the pS845/total GluA1/βIII-tubulin **(F)** and pS831/total GluA1/βIII-tubulin **(H)** ratios to that of the wild-type control samples. All data are displayed as mean ± SEM. Asterisks represent significance compared to wild-type control samples and the $ symbol indicates significant differences between the indicated categories. *p < 0.05, ^$^p < 0.05.

Overexpressed Caskin1 in cultured neurons was enriched in Shank2-positive dendritic spine heads (Fig. 7A) raising the possibility that Caskin1 can participate in the scaffold protein network of the PSD. Prominent changes in the amount of PSD scaffold proteins in Caskin dKO brain lysates, on the other hand, were not detected by western blotting as the normalized level of endogenous PSD-95 or Shank2, known members of the PSD, was similar between C57Bl6/J wild-type and Caskin dKO samples (Fig. 7B,C).

To check potential interactions between Caskin1 and Shank2 or PSD-95, co-IPs using Caskin1 or Shank2 antibodies were performed in WT and Caskin dKO brain lysates (Fig. 7D). Caskin1 antibody successfully pulled down Caskin1 and Shank2 from WT lysates, while Caskin1-specific signal was absent in Caskin dKO samples (Fig. 7D). Caskin1 antibody did not pull down PSD-95 in any of the tested lysates. On the other hand, Shank2 antibody co-precipitated Caskin1 from the WT lysates, indicating a potential interaction between endogenous Caskin1 and Shank2, the master scaffold protein of the PSD.

### Lack of Caskin proteins selectively alters AMPAR phosphorylation in basal as well as during cLTP-induced conditions

Phosphorylation of the GluA1 subunits can regulate targeting and stabilisation of AMPA receptors within the postsynaptic membrane^37,38^. Therefore, we used phospho-specific antibodies to detect phosphorylation of the S831 and S845 sites (pS831 and pS845) of the endogenous GluA1 subunit in Caskin dKO and C57Bl6/J (WT) cultures (Fig. 7E–H). Our data showed that in normal culture conditions, lack of Caskin proteins did not alter pS845 levels (Fig. 7E,F) but significantly elevated S831 GluA1 phosphorylation when compared to wild-type cultures (Fig. 7G,H).

As expected, phosphorylation at the S845 and S831 sites were significantly induced in wild-type hippocampal cultures by 5 hours following the 3 minute-long glycine treatment (cLTP; Fig. 7E–H). Elevation of the pS831 signal by cLTP was completely reverted by blocking NMDA receptors with APV (Fig. 7G,H). Interestingly, in Caskin dKO cultures, cLTP treatment did not alter the pS845 levels while relative pS831 phosphorylation in Caskin dKO hippocampal cultures was not increased further by cLTP treatment.

These data suggest that Caskin proteins selectively regulate the phosphorylation of GluA1 AMPA receptor subunits during basal as well as upon cLTP-induced conditions.

## Discussion

Caskin1 has been shown to mediate several protein-protein interactions and to impact animal behaviour^13,16,23^. To our knowledge, our work is the first to address Caskin-mediated effects on dendritic spine morphology and LTP formation. We have demonstrated that Caskin proteins regulate dendritic spine morphology *in vivo* and *in vitro*. Caskin proteins were shown to play a role in synaptic plasticity by regulating the extent of LTP and memory formation during novel object recognition and spatial navigation. Immunoprecipitation and immunocytochemistry analyses prove that Caskin1 and Shank2 co-localize within the dendritic spines indicating that Caskin1 can be a novel member of the postsynaptic scaffold protein network. Surprisingly, chemically induced morphological changes in dendritic spines are augmented by the absence of Caskin proteins. Additionally, site-specific GluA1 AMPA receptor subunit phosphorylation is selectively altered in Caskin dKO cultures, both under basal as well as after chemically induced LTP formation. Taken together, our data indicate that Caskin proteins have a substantial function in synaptic plasticity and memory formation, partly via regulating AMPAR localisation as well as activity-dependent morphological rearrangement of dendritic spines.

Previously, the Ito group reported the generation of Caskin1 KO mice^16^. Based on numerous animal behavioural tests, they concluded that Caskin1 plays a slight role in anxiety, pain perception, gait control and spatial memory formation in the Barnes maze. According to our results, deletion of both Caskins impaired spatial learning and object recognition, without severely affecting motor coordination. According to their published work, deleting Caskin1 evoked only minute changes in spatial memory after 1 month of the training sessions^16^. On the other hand, our Morris water maze results revealed that *i*) many of the tested dKO mice were unable to improve their performance during the 10 days of training or between the probe trials on day 5 and 10; *ii*) swimming towards the hidden platform was significantly less oriented in dKO mice; *iii*) trained dKO mice were less capable of finding the new position of the hidden platform. Even more prominent impairments were observed in case of performing the novel object recognition test, as deleting both Caskin proteins completely blocked their ability to recognize novelty in 5 hours.

Morris water maze is a widely used test to examine hippocampus-dependent spatial memory^39^ but the role of hippocampus in object recognition has remained unexplained^25,40^. Object recognition requires the perirhinal cortex^41^, the insular cortex^42^ and the ventromedial prefrontal cortex^43^ for memory encoding, storing and recovering. It is important to note that aged Caskin dKO mice showed significantly reduced exploration of the objects placed in their cage, regardless of whether these were new or familiar to them. The reduced exploration might originate from sensory deficits, especially when taking into account that Caskin1 was reported to be expressed in the bovine retina^17^. On the other hand, discrimination index was already severely impaired in young Caskin dKO mice, while the explorative behaviour in the open field test was identical to that of heterozygous littermates. Based on these observations, we assume that Caskin proteins play a general role in memory formation within different cortical regions already from young ages. Further investigations are needed to clarify the role of Caskin proteins in sensory perception.

It is also an intriguing question to which extent the published behavioural alterations of Caskin1 KO^16^ or our findings using dKO mice are due to the deletion of Caskin1 only or to the lack of both proteins. This is especially relevant as the deletion of Caskin1 was achieved by similar strategy in both studies. It is known that Caskin1 and 2 share similar N-terminal ankyrin, SH3 and SAM domains, while their C-terminal, proline-rich region shares only ~26% homology^13^. Despite their organizational similarity, Caskin2 is unable to bind to CASK, which is a proved synaptic partner of Caskin1^13^. Additionally, functional differences between the SAM as well as the SH3 domains have been also reported^14,15^. Thus, Caskin1 and 2 may have diverged with respect to their scaffolding functions in neurons, having different roles and protein partners^14,15^. Unfortunately, localisation and interactive partners of Caskin2 have not yet been identified and available experimental data address only its domain structure and polymerisation^14,15^. Therefore, we can not rule out that the more severe behavioural phenotype of the Caskin dKO mice is due to the absence of the Caskin2.

A dedicated role of Caskin2 might be related to the formation of dendritic protrusions, as protrusion density of cultured hippocampal neurons lacking both Caskin1 and 2 was significantly reduced in relation to wild-type dendritic branches and was not restored by overexpressing Caskin1. Additionally, activity-dependent changes in the density and morphology of dendritic spines seem to be independent from Caskin1. On one hand, cLTP evoked a significant increase in protrusion density but did not influence spine head expansion in dKO neurons. Moreover, these changes were unaffected by the overexpression or absence of Caskin1. These observations imply that the lack of Caskin2 might promote activity-dependent spine formation and/or morphological rearrangements. Further investigations are needed to clarify Caskin2-mediated effects in neurons.

Several sites of Caskin1, including its ankyrin repeats, the SH3 and SAM domains as well as the proline rich C-terminal region (PRD), have been suggested as potential interactive domains^13,20,44^. The PRD was reported to contain several consensus SH3 binding sites, mediating interactions with Nck^18^ or Abi2^20^. Nevertheless, Caskin1 and the SH3 domain containing PSD-95^45^ did not precipitate within the same complex. On the other hand, overexpressed Caskin1 colocalized with endogenous Shank2 in dendritic spine heads and co-precipitated with it. Although we have no direct evidence about the Caskin1 domain responsible for the interaction with Shank2, the master scaffold protein of the PSD^9^, SAM domains are the likely mediators of this protein-protein interaction^23^. Importantly, deletion of Caskin proteins resulted in distorted spine morphology and reduced PSD length within the hippocampus, while overexpression of Caskin1 increased the ratio of mushroom spines. These results indicate that Caskin1 likely regulate postsynaptic organization and spine head morphology.

While the size of the active zone and PSD length are mutually dependent^46^, the smaller presynaptic active zone revealed by ultrastructural analysis in dKO hippocampi argues for a potential presynaptic role of Caskins. The region between Caskin1’s SH3 and SAM domains is designated as the CASK-interaction domain (CID)^13,23^. Proposed models for Caskin1 mediated functions have already indicated a role in synaptic vesicle docking via CASK binding in the presynaptic area^23^ or regulating the binding of CASK to plasma membrane receptors in a competition with Mint-1^13^. On the other hand, potential presynaptic role of Caskin1 is questioned by the field potential measurements in acute hippocampal slices: deleting Caskin proteins did not alter basal excitability as the slope of the evoked EPSPs was similar to heterozygous slices before tetanization. Additionally, whole-cell voltage-clamp recordings in dissociated hippocampal neuronal cultures revealed that the frequency of the spontaneous neurotransmitter release was similar between wild-type, heterozygous or dKO neurons and was not changed upon Caskin1 overexpression in dKO cultures. It is important to note that spontaneous synaptic vesicle release differs in many aspects from the activity-dependent synaptic vesicle fusion^47,48^, so we can not rule out completely that Caskin proteins participate in presynaptic functions.

CASK has been reported to influence the delivery of AMPA and NMDA receptors as well as that of Kir2 channels to the synaptic membrane via regulating SAP97 interactions^49–52^. Caskins have been named after their CASK binding, implicating the possibility that Caskins also play a role in AMPA receptor trafficking and localisation. Indeed, our Western blot results provide clear evidence that site-specific phosphorylation of GluA1 subunits is selectively regulated by Caskin proteins during basal activity as well as following cLTP. S831 phosphorylation is known to play a role in GluA1 targeting to the PSD under basal conditions^37^ as well as in cLTP formation^53^, while phosphorylation of the S845 site has been implicated in several events, including the promotion of GluA1 delivery to the extrasynaptic surface, stabilisation of AMPA receptors and/or limiting endocytosis^54–56^. Importantly, relative pS381 GluA1 signal intensity was elevated in dKO neurons compared to wild-type cultures and were not increased further by cLTP. To the contrary, Caskin deficiency did not affect S845 site phosphorylation under basal conditions but completely eliminated cLTP-evoked changes in the relative pS845 GluA1 levels. These data indicate that Caskins attenuate phosphorylation of the S831 site under basal conditions while in case of LTP formation, Caskin proteins are required for promoting PKA-mediated S845 phosphorylation^56^. In agreement with this, high frequency stimulation evoked facilitation both in Caskin dHZ and dKO slices but dKO recordings had significantly lower POPS amplitude.

These data indicate that Caskin proteins play an important role in the activity-dependent relocation of AMPA receptors, needed for proper LTP and memory formation. Further investigations are needed to clarify how Caskin proteins regulate extrasynaptic plasma membrane delivery, synaptic targeting and localisation and/or activity-dependent turnover of AMPA receptors.

## Supplementary information

Supplementary Information File

Supplementary Dataset
