# Supplementary material for: Dendritic spine morphology and memory formation depend on postsynaptic Caskin proteins
Source: Sci Rep. 2019 Nov 14;9:16843. doi: 10.1038/s41598-019-53317-9 (PMC6856520; doi:10.1038/s41598-019-53317-9)
Supplement: Supplementary file 1 — Supplementary Information File [file 41598_2019_53317_MOESM1_ESM.docx]

**Dendritic spine morphology and memory formation depend on postsynaptic Caskin proteins**

Norbert Bencsik^1^, Szilvia Pusztai^1^, Sándor Borbély^1,2^, Anna Fekete^3^, Metta Dülk^3^, Viktor Kis^4^, Szabolcs Pesti^5^, Virág Vas^3^, Attila Szűcs^1^, László Buday^3,5^, Katalin Schlett^1*^

^1^Department of Physiology and Neurobiology, Eötvös Loránd University, Budapest, Hungary

^2^Institute of Cognitive Neuroscience and Psychology, Research Centre of Natural Sciences, Hungarian Academy of Sciences, Budapest, Hungary

^3^Institute of Enzymology, Research Centre of Natural Sciences, Hungarian Academy of Sciences, Budapest, Hungary

^4^Department Anatomy, Cell and Developmental Biology, Eötvös Loránd University, Budapest, Hungary

^5^Department Medical Chemistry, Semmelweis University, Budapest, Hungary

^*^corresponding author

**Supplementary Figure legends**

**Figure S1**

Western blot results obtained with three commercially available Caskin2 antibodies. None of the tested antibodies gave reliable signal at the expected molecular weight (~127 kDa) when C57Bl6/J wild-type (WT) and Caskin dKO liver, spleen, heart and brain lysates were compared.

**Figure S2**

Daily report on the number of unsuccessful Caskin dHZ and Caskin dKO mice during the Morris water maze test.

**Figure S3**

Interevent interval, amplitude and the decay time of mEPSCs recorded from DIV14-16 dissociated hippocampal neurons are similar, independently from Caskin expression levels. **(A-B)** Representative mEPSC traces (**A**) and mEPSC interevent intervals, amplitudes and the decay times **(B)** recorded from non-transfected C57Bl6/J wild-type (WT), Caskin dHZ and dKO neurons. **(C-D)** Representative mEPSC traces (**C**) as well as mEPSC interevent intervals, amplitudes and decay times (**D**) recorded from fluorescently labelled Caskin dKO hippocampal neurons, expressing either EGFP alone or EGFP in combination with V5-tagged Caskin1. Boxplots show the median (middle lines) and the 25-75 percentiles of the mEPSC data. Individual dots represent the median of a 200 sec-long mEPSC recording obtained from individual neurons. Data were obtained from 2-8 independent cultures; the number of measured cells is indicated above the graphs.

**Figure S4**

Comparison of EGFP-expressing wild-type hippocampal neurons obtained from C57Bl6/J or CD1 embryos. When dissociated neurons are cultured under identical conditions, they possess similar morphological characteristics of dendritic protrusions, as determined by Shank2 positivity and morphological classification. Data are presented as mean ± SEM. The number of analysed protrusions is indicated above the columns.

**Figure S5**

Complete images of the Western blot cut-outs presented in Figure 1E and 7B and D. Dashed lines show cropped ROIs from the corresponding blots.

**FigureS6**

Complete images of the Western blot cut-outs presented in Figure 7E and G. Dashed lines show cropped ROIs from the corresponding blots.
