# Supplementary material for: Dendritic spine morphology and memory formation depend on postsynaptic Caskin proteins
Source: Sci Rep. 2019 Nov 14;9:16843. doi: 10.1038/s41598-019-53317-9 (PMC6856520; doi:10.1038/s41598-019-53317-9)

Dendritic spine morphology and memory formation depend on postsynaptic Caskin proteins

Norbert Bencsik, Szilvia Pusztai, Sándor Borbély, Anna Fekete, Metta Dülk, Viktor Kis, Szabolcs Pesti, Virág Vas, Attila Szűcs, László Buday, Katalin Schlett

Figure S1

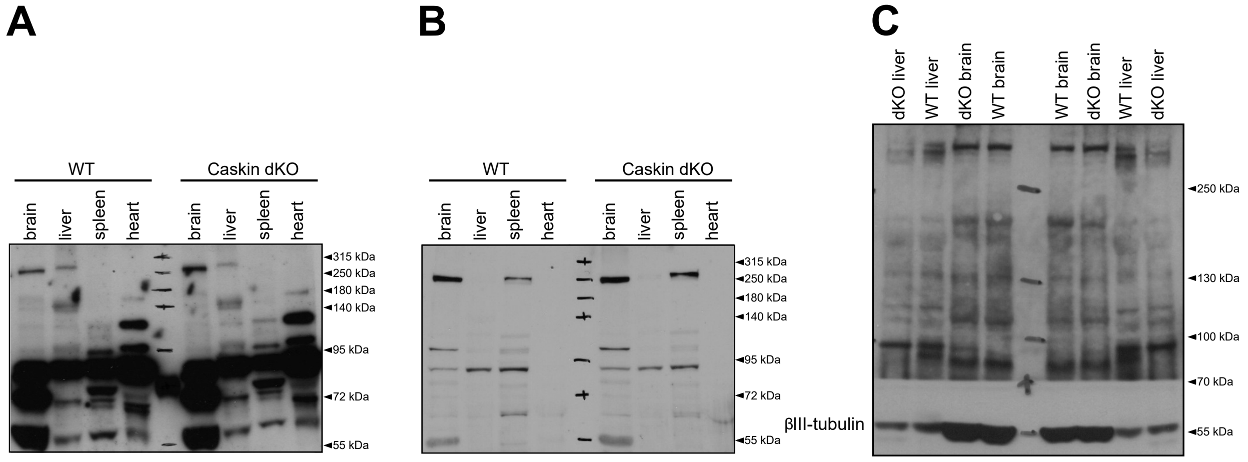

manufacturer:	AbCam	ThermoFisher	Santa Cruz
description:	polyclonal rabbit	polyclonal rabbit	monoclonal mouse
specifity:	714-741 aa	714-741 aa	382-407 aa
catalogue number:	ab107421	PA5-24430	sc-393825

Dendritic spine morphology and memory formation depend on postsynaptic Caskin proteins

Norbert Bencsik, Szilvia Pusztai, Sándor Borbély, Anna Fekete, Metta Dülk, Viktor Kis,
Szabolcs Pesti, Virág Vas, Attila Szűcs, László Buday, Katalin Schlett

Figure S2

A

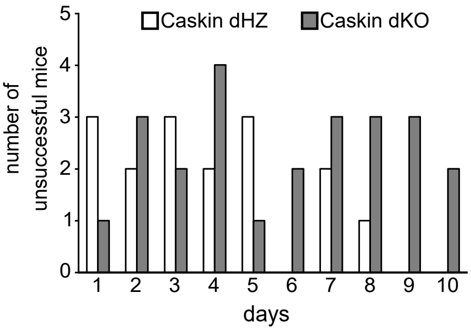

Dendritic spine morphology and memory formation depend on postsynaptic Caskin proteins

Norbert Bencsik, Szilvia Pusztai, Sándor Borbély, Anna Fekete, Metta Dülk, Viktor Kis, Szabolcs Pesti, Virág Vas, Attila Szűcs, László Buday, Katalin Schlett

Figure S3

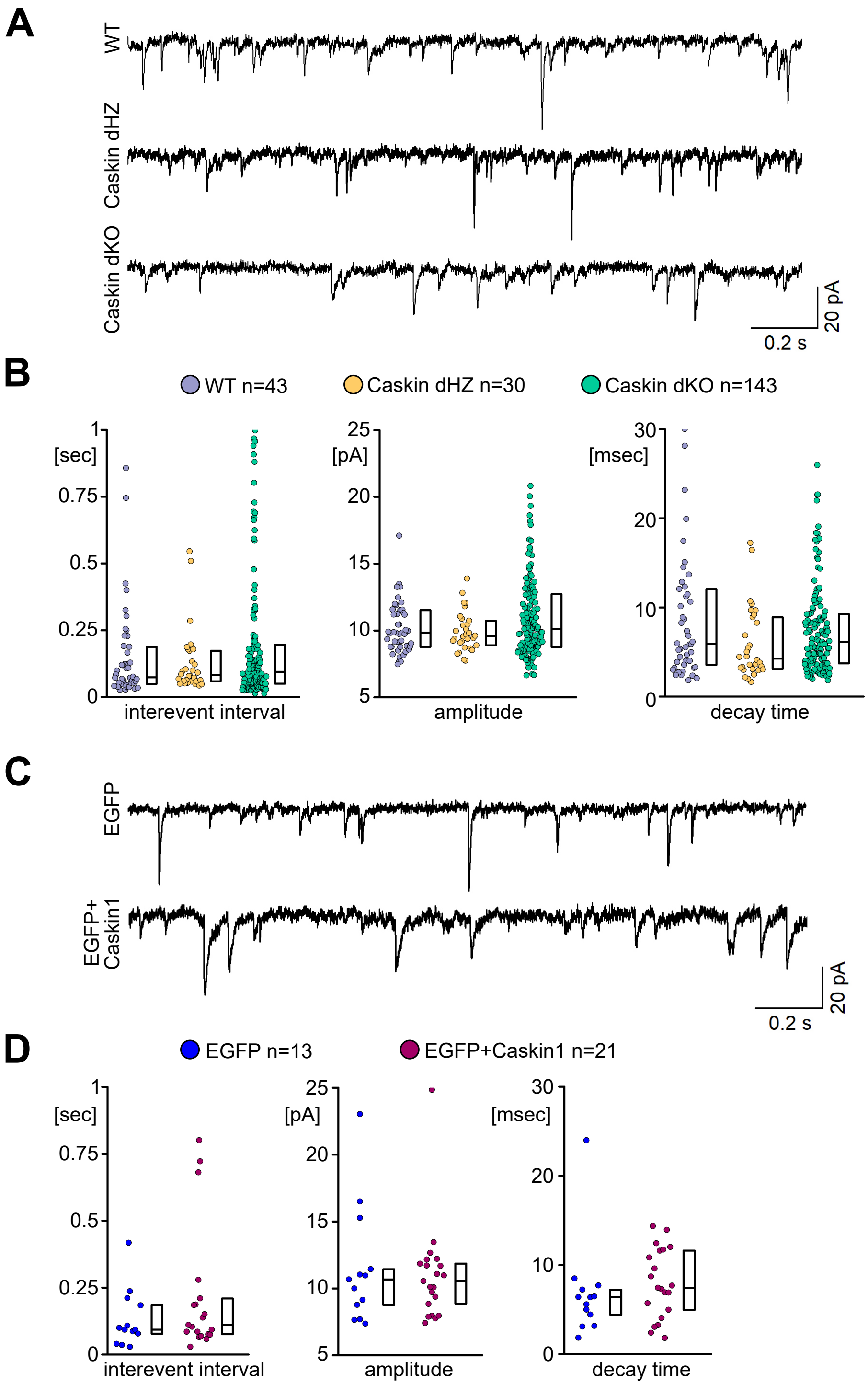

Dendritic spine morphology and memory formation depend on postsynaptic Caskin proteins

Norbert Bencsik, Szilvia Pusztai, Sándor Borbély, Anna Fekete, Metta Dülk, Viktor Kis, Szabolcs Pesti, Virág Vas, Attila Szűcs, László Buday, Katalin Schlett

Figure S4

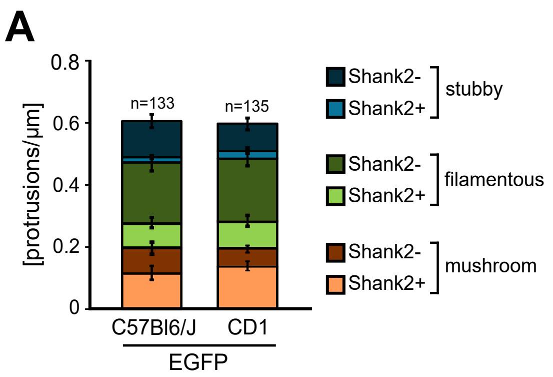

Dendritic spine morphology and memory formation depend on postsynaptic Caskin proteins

Norbert Bencsik, Szilvia Pusztai, Sándor Borbély, Anna Fekete, Metta Dülk, Viktor Kis, Szabolcs Pesti, Virág Vas, Attila Szűcs, László Buday, Katalin Schlett

Figure S5

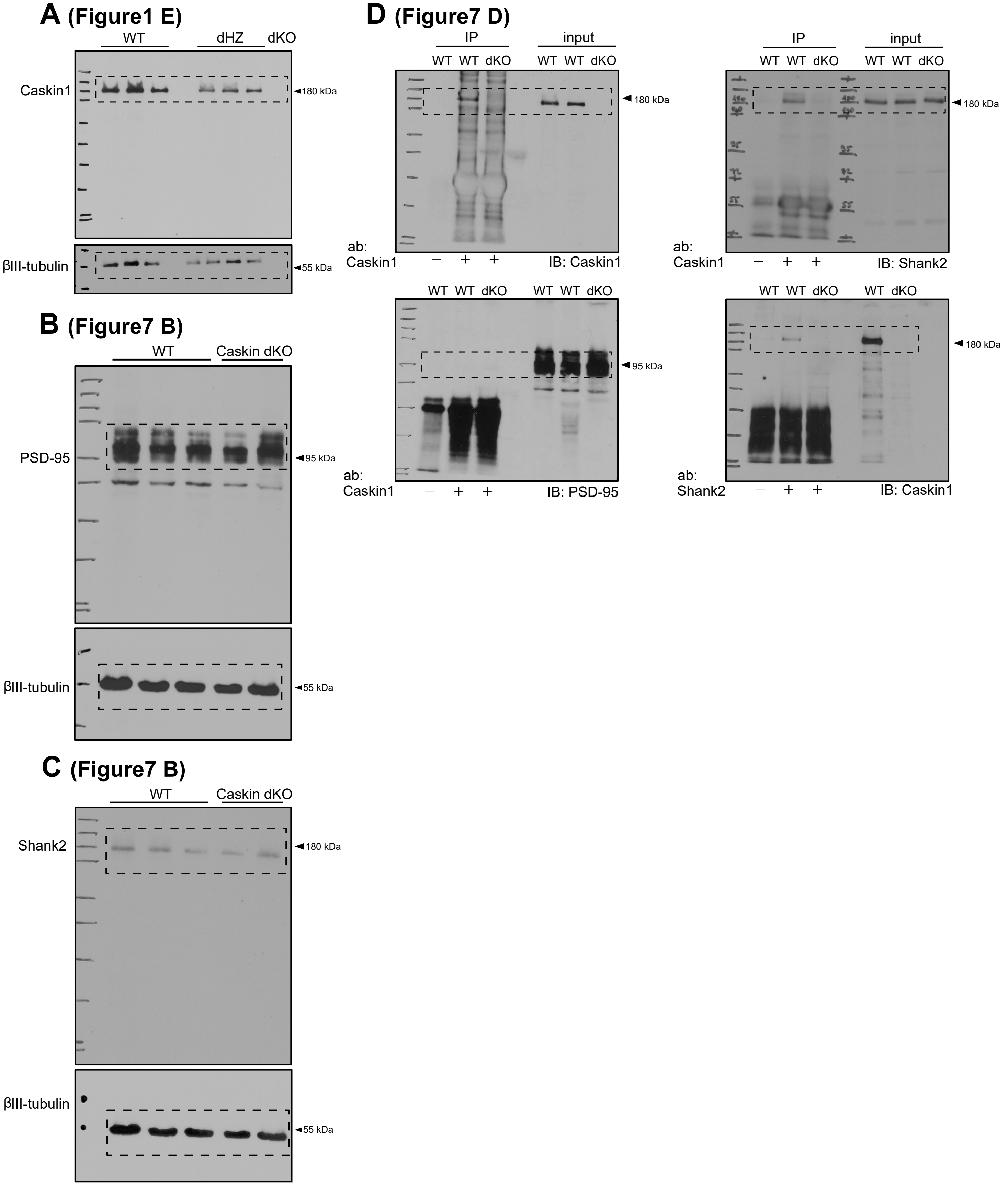

Dendritic spine morphology and memory formation depend on postsynaptic Caskin proteins

Norbert Bencsik, Szilvia Pusztai, Sándor Borbély, Anna Fekete, Metta Dülk, Viktor Kis, Szabolcs Pesti, Virág Vas, Attila Szűcs, László Buday, Katalin Schlett

Figure S6

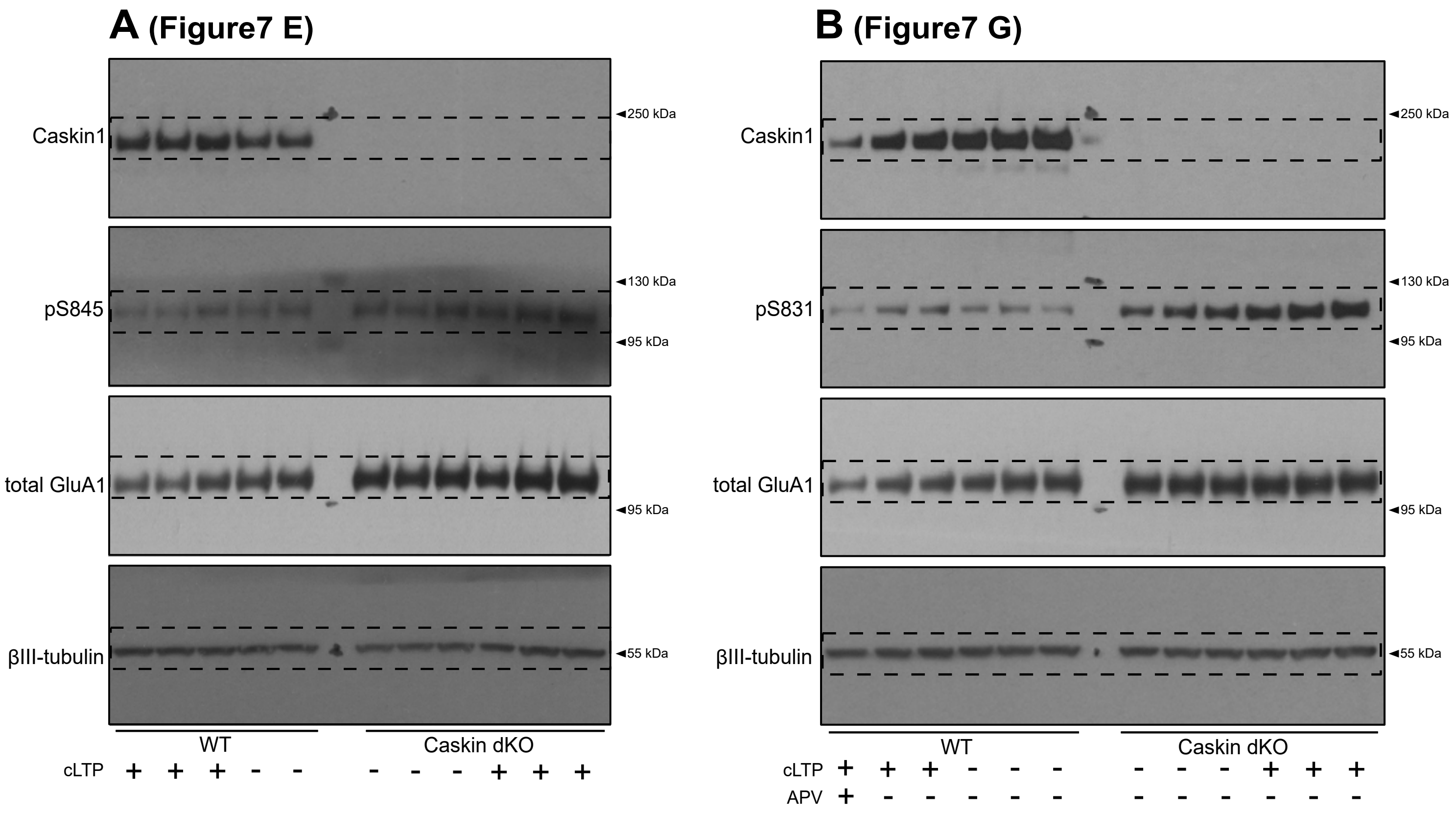

Supplement: Supplementary file 2 — Supplementary Dataset [file 41598_2019_53317_MOESM2_ESM.pdf]
